# Detection of Asherman’s syndrome after conservative management of placenta accreta: a case report

**DOI:** 10.1186/s13256-018-1869-7

**Published:** 2018-11-20

**Authors:** Kenro Chikazawa, Ken Imai, Wang Liangcheng, Shigetane Sasaki, Isao Horiuchi, Tomoyuki Kuwata, Kenjiro Takagi

**Affiliations:** Perinatal Center, Division of Maternal Fetal Medicine, Saitama Medical Center, Jichi Medical University, 1-847, Amanuma-cho, Omiya-ku, Saitama, 330-8503 Japan

**Keywords:** Placenta accreta, Conservative management, Asherman’s syndrome

## Abstract

**Background:**

We present a case involving conservative treatment of placenta accreta, with a subsequent diagnosis of Asherman’s syndrome.

**Case presentation:**

A 41-year-old Japanese woman (G2P0A2) delivered a healthy male infant via cesarean section due to preeclampsia. The placenta did not spontaneously separate and was manually removed. Adhesion was tight and placenta accreta was diagnosed. During the procedure, no uterine inversion or perforation, and no uterine cavity adhesion, were observed. Four months postoperatively, hysteroscopy was performed. Adhesion was detected at the fundus of her uterus where the placenta had adhered to the uterus. Asherman’s syndrome was diagnosed.

**Conclusions:**

Asherman’s syndrome might occur after conservative management of placenta accreta, which may be a direct cause of placenta accreta recurrence. When Asherman’s syndrome is diagnosed, the site of the placenta and adhesion should be monitored during subsequent pregnancies.

## Background

Several recent reports have discussed the conservative management of placenta accreta [1, 2]; however, no established method currently exists. Moreover, the management of subsequent pregnancies after conservative treatment of placenta accreta has not been established. The recurrence rate of placental attachment disorder during subsequent pregnancies after conservative management of placenta accreta was reported to be 20% [3]. Given this high recurrence rate, the ability to predict the recurrence of placenta accreta would be useful.

Asherman’s syndrome is associated with the risk of placenta accreta; out of all cases of Asherman’s syndrome, 12.5% experience postpartum hemorrhage due to placenta accreta [4]. Here we present a case involving conservative treatment of placenta accreta, with a subsequent diagnosis of Asherman’s syndrome. The present case suggests that, after conservative treatment of placenta accreta, patients may progress to Asherman’s syndrome. Thus, Asherman’s syndrome following conservative treatment of placenta accreta might be a direct cause of placenta accreta recurrence.

## Case presentation

A 41-year-old Japanese woman (gravida 2, para 0) had two previous miscarriages during the first trimester. She became pregnant via *in vitro* fertilization. Ultrasound findings during the second and third trimesters were not indicative of placenta accreta. She developed preeclampsia during the 36th week of gestation and underwent caesarean section. She delivered a healthy male infant (2178 g) with Apgar scores of 8 and 9 at 1 and 5 minutes, respectively. However, the placenta did not spontaneously separate; thus, the operator separated and gently removed the placenta from the uterine cavity manually. As adhesion was tight, placenta accreta was diagnosed. During the procedure, no uterine inversion or perforation was observed and there were no uterine cavity adhesions, such as those in Asherman’s syndrome. Manual removal was successfully performed.

Continuous bleeding was observed after removal of the placenta; thus, uterine gauze packing was performed, and the bleeding was stopped. On postoperative day 1, there was little bleeding; thus, the obstetrician removed the gauze. However, severe bleeding reoccurred. A balloon (Bakri® Balloon, Tokyo, Japan) was inserted into her uterine cavity and the bleeding stopped again. On postoperative day 3, the balloon was removed and there was no active bleeding this time.

**Fig. 1:**
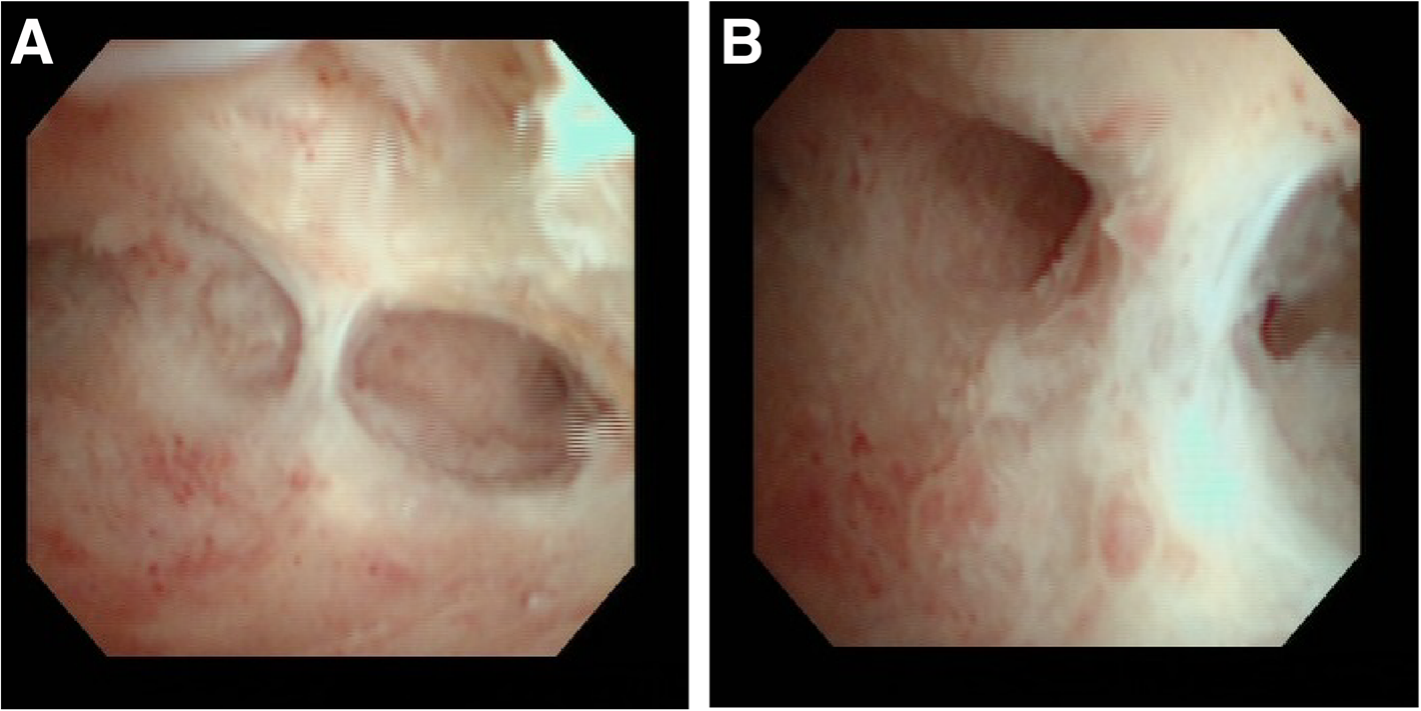
Post-treatment hysteroscopy detected adhesion at the fundus of the uterus. **a** Distant view, **b** close view

One month postoperatively, she had no abnormal complaints. Two months postoperatively, her menses restarted. Four months postoperatively, we performed hysteroscopy. We detected an adhesion at the fundus of her uterus, in the location of the placenta accreta (Fig. 1). Asherman’s syndrome was diagnosed.

## Discussion and conclusions

The present case indicates that Asherman’s syndrome may occur after the conservative treatment of placenta accreta. The site of adhesion in the uterine cavity matched the location of the placenta accreta. Thus, Asherman’s syndrome after the conservative treatment of placenta accreta might be a direct cause of placenta accreta recurrence.

The present study is the first to report hysteroscopic observations soon after the resumption of menses after the conservative treatment of placenta accreta. Although some reports exist regarding direct treatment with hysteroscopy for placenta accreta [5, 6], there are no previous reports of hysteroscopic observations following conservative treatment alone. The present case suggests that Asherman’s syndrome may occur even without invasive uterine treatment. Legendre *et al.* [6] reported that, among four cases that underwent conservative treatment for placenta accreta, one ectopic pregnancy and one miscarriage occurred. Thus, conservative treatment for placenta accreta might be a cause of adhesions in the uterine cavity. Roy *et al*. [4] reported that the conception rate in patients with Asherman’s syndrome was 30–58%, with a miscarriage rate of 11.1%. Considering these findings, undiagnosed Asherman’s syndrome may exist after conservative treatment of placenta accreta, which may be a cause of placenta accreta recurrence after conservative treatment.

Severe postpartum hemorrhage due to placenta accreta has been observed in Asherman’s syndrome [4]. In addition, 13% of cases with placenta accreta have a history of Asherman’s syndrome [7]. The detection of abnormal findings in the uterine cavity on hysteroscopy after conservative treatment of placenta accreta would indicate the importance of considering the site of the placenta and adhesion in subsequent pregnancies. Conventional two-dimensional ultrasonography is useful in screening for placenta accreta, with both sensitivity and specificity over 90%, and a negative predictive value of 98% [8–10]. Patients with a medical history of Asherman’s syndrome after conservative management of placenta accreta should be examined by a skilled sonographer, who should be vigilant for the recurrence of placenta accreta. In addition, such patients should be managed in a critical care medical center with skilled clinicians, given the risk of severe postpartum hemorrhage.

This case report had a limitation. There were no data on pre-pregnancy hysteroscopy. Thus, we did not know whether the adhesion existed before *in vitro* fertilization.

In conclusion, Asherman’s syndrome might occur after conservative management of placenta accreta, which may be a direct cause of recurrence. Observations by hysteroscopy are useful for arriving at the correct diagnosis. When Asherman’s syndrome is diagnosed, the site of the placenta and adhesion should be monitored during subsequent pregnancies.
